# How does mass loss compare with total body score when assessing decomposition of human and pig cadavers?

**DOI:** 10.1007/s12024-022-00481-6

**Published:** 2022-05-11

**Authors:** Blake M. Dawson, James F. Wallman, Philip S. Barton

**Affiliations:** 1 Centre for Sustainable Ecosystem Solutions, School of Earth, Atmospheric and Life Sciences, University of Wollongong, Wollongong, NSW Australia; 2 Faculty of Science, University of Technology Sydney, Ultimo, NSW Australia; 3 Future Regions Research Centre, Federation University Australia, Mount Helen, VIC, Australia

**Keywords:** Cadaver, Carrion, Post-mortem interval, Taphonomy

## Abstract

Providing accurate and reliable measures of decomposition is paramount for forensic research where decomposition progress is used to estimate time of death. Mass loss is routinely used as a direct measure of biomass decomposition in ecological studies, yet few studies have analysed mass loss in a forensic context on human cadavers to determine its usefulness for modelling the decomposition process. Mass loss was examined in decomposing human and pig cadavers, and compared with other common decomposition metrics, such as total body score (*TBS*). One summer and one winter field decomposition experiment was conducted using human and pig cadavers, as pigs are often used as proxies for human cadavers in forensic research. The two measures of decomposition revealed two contrasting patterns of decomposition on pigs and humans, particularly in winter where *TBS* stabilised at similar values, but mass loss differed greatly. Mass loss was found to be faster in pigs than humans during early decomposition. Pigs lost 75% of their mass in winter, while humans lost less than 50%; however, in summer, both lost around 80% of their mass. *TBS* displayed similar patterns in both experiments, with *TBS* increasing more rapidly in pigs compared with humans but both eventually reaching similar *TBS* values in late decomposition. Measuring mass loss can provide additional information about decomposition progress that is missed if using *TBS* only. Key differences in decomposition progress between cadaver types were also observed, suggesting caution when extrapolating data from pigs to humans for forensic research and decomposition modelling.

## Introduction

Decomposition is a natural process whereby organic matter is broken down and consumed, releasing a pulse of nutrients back into the local environment [1]. Knowledge of the decomposition process can aid forensic investigators in estimating a post-mortem interval (PMI), which is the minimum and maximum amount of time for which an individual might have been deceased [2]. Decomposition is a highly variable process and is influenced by numerous factors such as ambient temperature, habitat, carrion mass, scavenger activity, and microbes [3–8]. Due to the variable nature of decomposition, determining a universal and standardised metric for quantifying the decomposition process for forensic (and ecological) purposes has been challenging [9].

Traditionally, the decomposition process has been divided into distinct categorical decomposition stages based on visual morphological changes in the soft tissue of remains, such as the onset of bloat or bone exposure [10]. The advent of decay “stages” enabled other post-mortem processes, like insect activity, to be temporally linked to decomposition, thereby providing new sources of PMI estimation [11]. Although these decay stages were fundamental in providing the groundwork for categorising decomposition, they have several associated issues. First, numerous studies have used different numbers of decay stages, with no universally accepted number of such decay stages [10], and second, decomposition is a continuous process and should therefore be treated as a continuum [12].

The total body score (*TBS*) metric was developed to improve upon the decay stage approach and provide a semi-quantitative measure of decomposition [2]. This method separates a cadaver into three distinct regions (head, torso and limbs) and provides each region with a numeric value again based on the visual changes occurring during decomposition [2]. The scores from each region are summed together to provide a numeric value representing decomposition progress. *TBS* can be compared with temporal measures of time or other measures incorporating time and temperature (accumulated degree days, *ADD*) to provide a model-based approach for PMI estimation [13]. The reliability of this method to produce accurate PMI estimates has been questioned [14, 15], which has led to the development of more complex *TBS* models to improve accuracy and reliability [9, 16]. Though *TBS* more accurately reflects decomposition progress than decay stages, it is still an indirect measure of decomposition, being based on morphological changes rather than any quantifiable ones, such as chemical, microbial, or physiological changes in the cadaver [17, 18].

A direct measure of decomposition needs to incorporate the bio-physical changes occurring in a cadaver. As a cadaver decomposes, biomass is lost via fluid leakage into the soil, desiccation and consumption by organisms, eventually leading to complete mass loss once the skeleton breaks down [1, 19]. Mass loss during decomposition is a continuous process and an ideal metric that directly reflects decomposition progress [20, 21]. However, collecting mass loss data can be difficult as most studies rely on small-bodied non-human animal cadavers, as periodically weighing large human or other animal cadavers can be logistically challenging and may disturb the decomposition process and associated entomological activity [4, 22]. Despite this, mass loss has often been used in ecological research as a means of quantifying the decomposition process and to determine how nutrients and biomass is redistributed back into the local environment (4, 19, 23, 24). However, in a forensic context, mass loss is not often used as a measure of decomposition, or incorporated into PMI calculations. The few forensic studies that do analyse mass loss are often conducted on animal models, of which the domestic pig is the most common [25–27]. Mass loss in human cadavers has seldom been studied, and to date, no research has been undertaken on comparing mass loss between pigs and humans.

In this study, mass loss was examined in decomposing humans and pigs over the course of one winter and one summer field decomposition experiment. Mass loss was compared with another measure of decomposition, *TBS*, to determine what similarities or differences occur among the different measures of decomposition. A novel field method was also developed for weighing cadavers and collecting mass loss data during the decomposition process for large-bodied vertebrate remains. Both human and pig remains were used in this study as pigs are often used as proxies for humans in forensic research [28, 29]. Both cadaver types were assessed here to determine whether decomposition progress showed similar patterns between the two, which may be important for interpreting *TBS* or mass loss data when estimating the PMI or other aspects of decomposition [29].

## Material and methods

### Study site

One winter experiment (8th May to 2nd October 2019) and one summer experiment (9th November to 16th December 2019) were conducted at the Australian Facility for Taphonomic Experimental Research (AFTER), a 4.86 ha site located in the Hawkesbury region of Sydney, Australia. The facility is operated by the University of Technology Sydney (UTS) and allows for human decomposition to be examined in a natural environment. The local vegetation in the facility is dominated by dry sclerophyll *Eucalyptus* forest with scattered urban housing in the nearby vicinity.

### Human and pig cadavers

In the winter experiment, two male human donors (Human 1: age 57, 66 kg, placed 8/5/19 and Human 2: age 74, 51.7 kg, placed 7/6/19) and two female pigs (Pig 1: age 4–6 months, 70.6 kg, placed 8/5/19 and Pig 2: age 4–6 months, 57.7 kg, placed 11/6/19) were used, while the summer experiment consisted of two female humans donors (Human 3: age 82, 60.5 kg, placed 9/11/19 and Human 4: age 97, 46.8 kg, placed 14/11/19) and two female pigs (Pig 3: age 4–6 months, 102.9 kg, placed 11/11/19 and Pig 4: age 4–6 months, 63.5 kg, placed 18/11/19). Human donors were obtained through the UTS Body Donation Program, approved by the UTS Human Research Ethics Committee Program Approval (UTS HREC REF NO. ETH15-0029). Domestic pigs (*Sus scrofa*) were purchased post-mortem from a licensed abattoir, therefore requiring no ethics approval in accordance with the Australian Code of Practice for the Care and Use of Animals for Scientific Purposes (2004). Pigs were euthanised by a captive head bolt and transported to AFTER within 1 h of death, while humans were delivered to AFTER within 48 h of death and kept refrigerated for the duration of transportation.

Once at AFTER, all human cadavers were placed on their backs in 5 × 5 m plots within the facility. Pig carcasses were placed on their sides along the outside of the facility to comply with licencing agreements. Pigs were placed a minimum of 100 m away from the human cadavers and a minimum of 20 m from one another. Within the facility, human cadavers were placed at least 30 m apart. Thin metal mesh was also placed beneath each pig and human to enable the lifting and weighing of remains throughout decomposition. A scavenger proof cage was placed over each pig and human to prevent scavenging by larger animals. A HOBO MX2302 Ext logger with a solar radiation shield was placed on site to record ambient temperature and humidity every 15 min throughout both experiments.

### Measuring decomposition

#### Direct mass loss

**Fig. 1:**
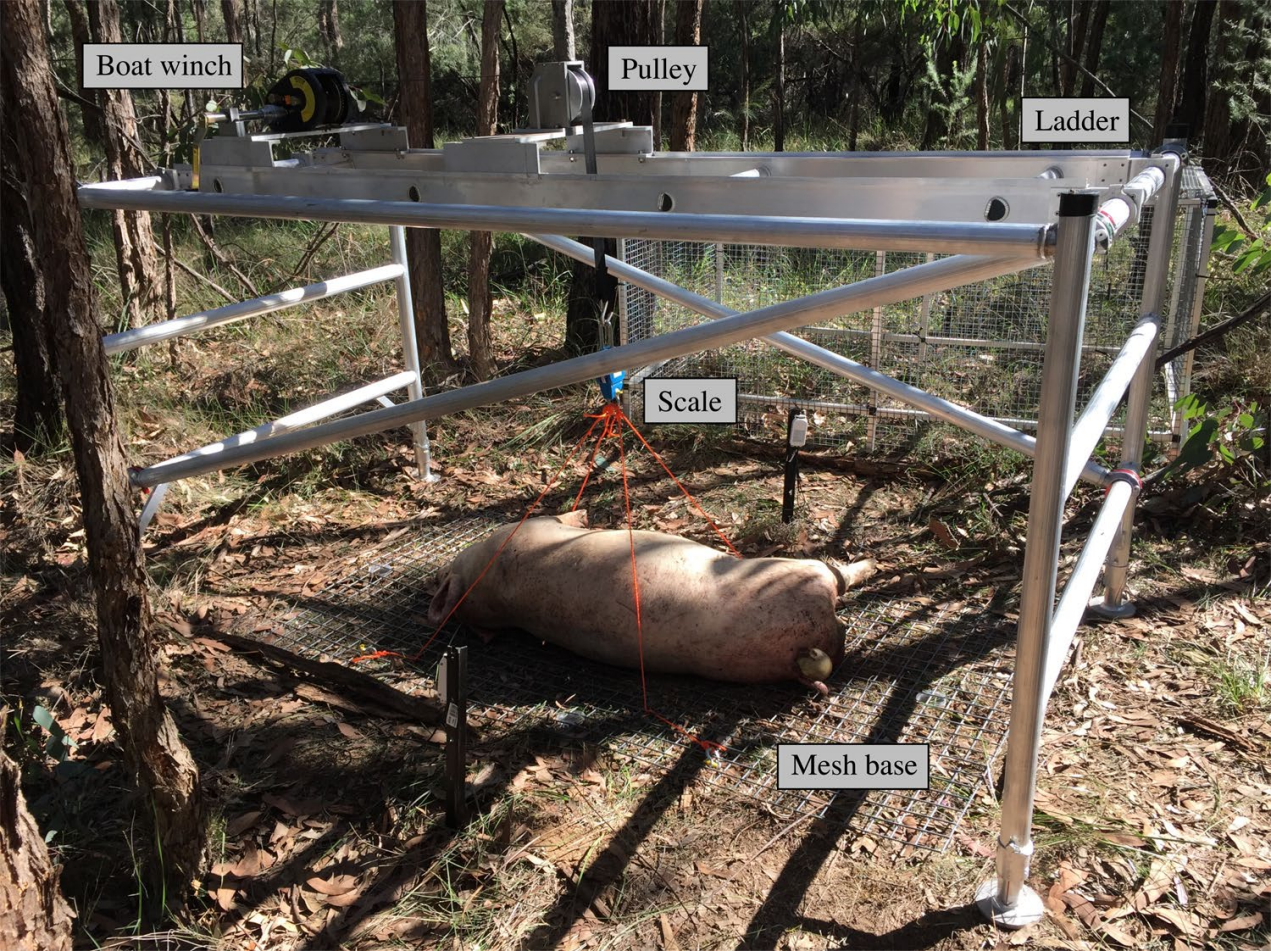
Modified scaffold unit set up over a decomposing pig to measure mass loss

To measure mass loss, a single aluminium scaffold unit with a platform ladder (2.5 m length × 1.3 m width) was converted into a lightweight, mobile weighing mechanism (Fig. 1). First, the scaffold unit was altered to remove the outer platform, leaving only the ladder which runs along the top of the scaffold connecting the two ends. A pulley system was attached to the ladder by attaching a boat winch (Jarrett Trailer Winch) onto one end of the ladder. The winch wire was replaced with a polyester belt and fed into a metal pulley, which was welded to the centre of the ladder. This allowed the mass of the cadavers to be centred in the middle of the scaffold. At the end of the polyester belt, a small hook was attached, allowing for a digital hanging scale (Wedderburn, 150 kg) to be connected.

Sampling occurred every 2–3 days during the first 1–2 weeks while decomposition progressed rapidly. Once decomposition slowed, sampling occurred every 5–7 days until the experiment was concluded. For the winter experiment, all individual pigs and humans were sampled a total of 11 times each. For the summer experiment, sampling intensity varied between replicates with a range of 3–6 measurements taken per replicate. This discrepancy was due to the quick rate of decomposition observed in summer with skeletisation occurring in one pig carcass within 8 days.

On each sampling day, the scaffold unit was placed directly over a cadaver and four hooks connected to rope were attached to the corners of the wire mesh, while the other end of the rope was attached to the hanging scale. Using the lever, the mesh was lifted slightly above the ground (approximately 2 cm) for no more than 10 s to record mass loss. The mesh had also been weighed prior to cadaver placement so the cadaver mass could be determined by subtracting the mesh mass from the sampling day mass.

#### Total body score

Decomposition progress was visually assessed every sampling day using the *TBS* method of Megyesi et al. [2]. The cadavers were divided into three distinct body regions (head, torso and limbs) and provided with a numeric score representing decomposition progress. These scores were summed together to provide a numeric value (*TBS*) for the total decomposition progress.

### Data analysis

Linear regression models were used to determine relationships between mass loss and *TBS* with the post-mortem interval (PMI) [16]. For PMI, accumulated degree days (*ADD*) were used, which were calculated by determining the average ambient temperature each day and cumulatively summing them together. Three different decomposition metrics were used to compare against *ADD*: mass loss, *TBS*, and adjusted *TBS*. For adjusted *TBS*, *TBS* was transformed using the formula: *TBS*_adj_ = *TBS* − 3 and compared against log*ADD* to create a more linear relationship between *TBS* and *ADD* (adapted from Moffatt et al. [16]). Values equal to 0 *ADD* were removed from the adjusted *TBS* analysis as log transforming these data was not possible. Mass loss was converted into a proportion of initial mass to standardise among the different starting masses of the cadavers. An additional linear regression model was also constructed comparing mass loss to *TBS*. Four separate linear models were constructed for each cadaver type, resulting in a total of eight models. Regression models and *R*-squared values were visually compared to assess how much variation in the model is explained by the independent variable [30]. All regression models were conducted using the R base package [31] and lme4 package [32], while plots were created using the ggplot2 package [33].

## Results

### Winter

**Fig. 2:**
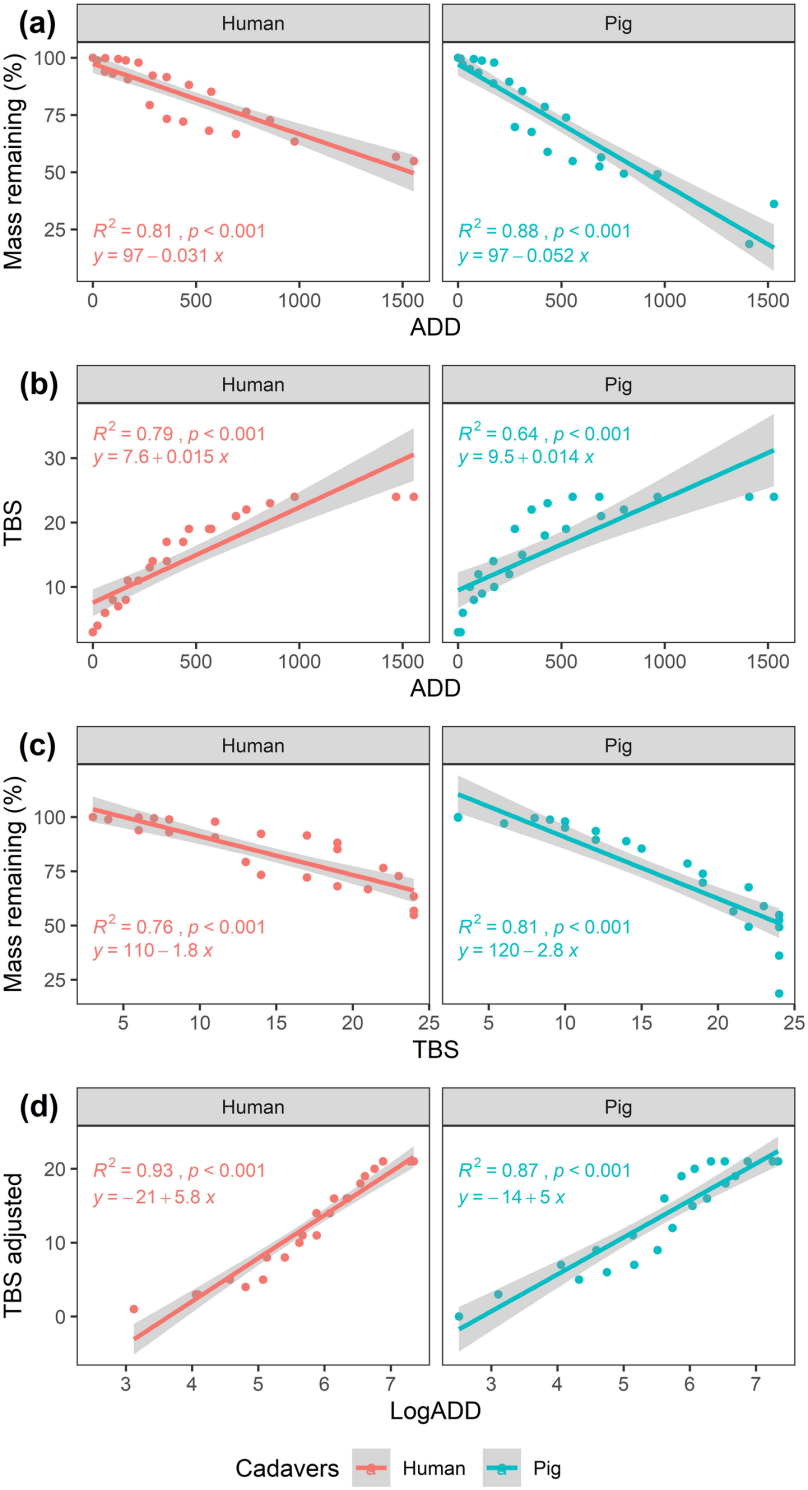
Decomposition progress measured as **a** mass remaining (%) compared against accumulated degree days (*ADD*), **b** total body score (*TBS*) compared against *ADD*, **c** mass remaining (%) compared against *TBS*, and **d** adjusted *TBS* (*TBS*_adj_ = *TBS* − 3) compared against *ADD* for human (red) and pig (blue) cadavers in the winter experiment. Grey bands represent SE

Mass loss was found to be significantly negatively correlated with *ADD* for both humans and pigs (Fig. 2a). Overall, pigs lost more mass than humans, with pigs reaching around 75% mass loss while humans lost no more than 50% of their mass. *TBS* was also found to be significantly positively correlated with *ADD* for humans and pigs (Fig. 2b). Unlike the humans, *TBS* values rose rapidly on pigs, reaching above 20 *TBS* before plateauing. Despite the more rapid rate of *TBS* increase on pigs, humans also eventually plateaued at the maximum recorded *TBS* of 24. When comparing mass loss to *TBS*, humans and pigs were found to be significantly negatively correlated (Fig. 2c). Mass loss occurred at a faster rate in pigs with around 25% of the mass loss occurring at 24 *TBS*. The adjusted *TBS* models were also positively significantly correlated with *ADD* with high *R*-squared values (Fig. 2d). In general, pigs displayed more unexplained variation in the data, with lower *R*-squared values and more measurements lying outside the SE range compared with humans.

### Summer

**Fig. 3:**
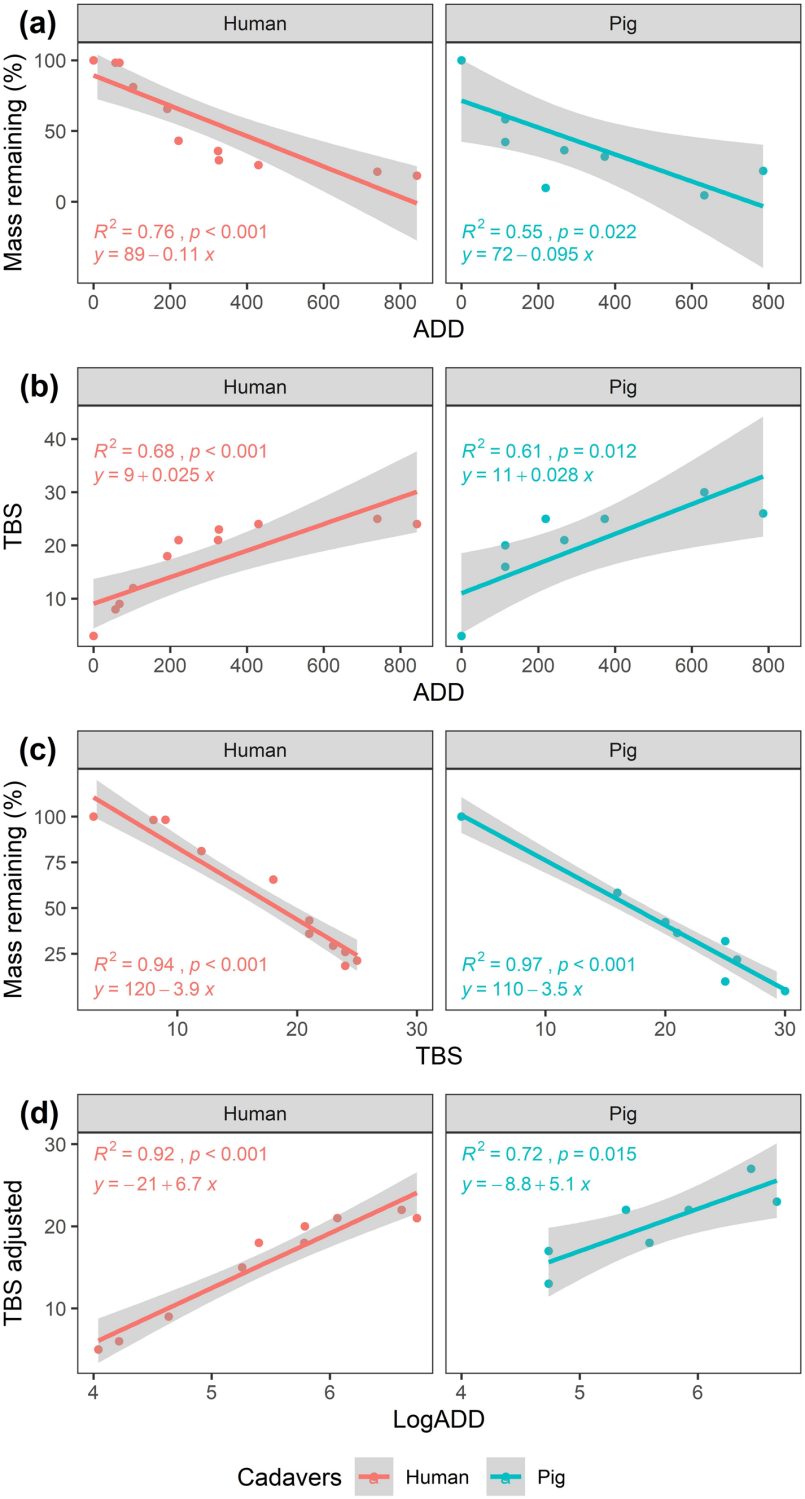
Decomposition progress measured as **a** mass remaining (%) compared against accumulated degree days (*ADD*), **b** total body score (*TBS*) compared against *ADD*, **c** mass remaining (%) compared against *TBS*, and **d** adjusted *TBS* (*TBS*_adj_ = *TBS* − 3) compared against *ADD* for human (red) and pig (blue) cadavers in the summer experiment. Grey bands represent SE

In summer, mass loss was found to be significantly negatively correlated with *ADD* for humans and pigs (Fig. 3a). For the first 100 *ADD*, pigs lost roughly 50% of their mass, while humans did not reach 50% mass loss until around 200 *ADD*. Despite this initial mass loss difference, both humans and pigs reached around 80–90% mass loss by the end of their decomposition. *TBS* was found to be significantly positively correlated with *ADD* for both humans and pigs (Fig. 3b). *TBS* exhibited a similar pattern to the mass loss model with *TBS* initially increasing rapidly on pigs to 20 *TBS* at 100 *ADD*, while humans did not reach the same *TBS* until 200 *ADD*, but both reached similar *TBS* values by the end of their decomposition. Mass loss compared with *TBS* was significantly negatively correlated with a surprisingly strong linear relationship for humans and pigs, as most of the variation in the model was explained with a *R*-squared of 0.94 and 0.97, respectively (Fig. 3c). Adjusted *TBS* was found to also be significantly positively correlated with *ADD* humans and pigs. More variation in the human model was explained relative to the pig model with a *R*-squared of 0.92 for humans and 0.72 for pigs (Fig. 3d).

## Discussion

Mass loss was measured in decomposing humans and pigs, and compared with a commonly used but indirect measure of decomposition, *TBS*. The results indicated variation in mass loss patterns between seasons and cadaver types. At the beginning of decomposition, pigs lost mass more rapidly than humans in both experiments. In winter, pigs had lost more mass than humans by the end of decomposition, but in summer, mass loss was similar between humans and pigs by the end of their decomposition. *TBS* was also found to increase more rapidly in pigs compared with humans at the start of decomposition, but both eventually plateaued and reached similar *TBS* by the end of decomposition during both seasons. Notably, mass loss had a strong linear relationship with *TBS* in summer when decomposition was more rapid, but in winter, mass loss progressed more slowly with a large portion of mass loss occurring at high *TBS* values, particularly for pigs. This study provides new insight into mass loss and *TBS* patterns in pigs and humans, highlighting how they represent different aspects of decomposition. These results also provide the first mass loss benchmark for *TBS* observations for both human and pig cadavers in warm and cool weather conditions.

### Mass loss between cadaver types

Pigs lost mass quicker than humans in both experiments, and by the end of the winter experiment, pigs had lost 75% of their mass while humans had lost 50%, but by the end of the winter experiment, both pigs and humans had lost 80–90% of their mass. This suggests that the decomposition progress and rate were different between the two cadaver types, which is similar to previous research comparing cadaver types using *TBS* [34–37]. Mass loss is driven by environmental factors (desiccation via evaporation) and organisms consuming and dispersing the remains [19]. Humans and pigs were placed around the same time and experienced similar ambient temperatures; therefore, environmental desiccation rates would likely also have been similar between cadaver types. Insect activity, however, was different between the cadaver types, as previous research using the same cadavers as this experiment showed differences in species richness of insects, particularly in Diptera between pigs and humans [38]. Insects have the ability to remove a significant amount of biomass from decomposing remains and therefore, are likely one of the key reasons why mass loss was different between pigs and humans [11].

The anatomy and physiology of humans and pigs have often been stated to be similar in terms of skin composition, fat-to-muscle ratio, and proportional organ size. Despite this, there are some differences between pigs and humans that may have contributed to total mass loss differences observed in this study, particularly water content (humans often have a higher percentage than pigs) and the condensed body structure of pigs [37, 39, 40]. However, total mass loss was only different in the winter experiment; therefore, if body composition was influencing mass loss, we would expect similar results in summer. It is likely mass loss rates were driven by other factors, such as insect activity or potentially microbial activity and the peri-mortem treatment of the cadavers [1, 36, 37].

### Mass loss compared with TBS

*TBS* exhibited similar patterns to mass loss when compared with *ADD*, displaying a more rapid increase in *TBS* on pigs at the start of decomposition. Both measures of decay accurately reflect the more rapid decomposition progress in pigs compared with humans at the start of start of decay. However, in both experiments, *TBS* plateaued at 24 *TBS* on humans and pigs for roughly 500 *ADD* at the end of decomposition. Over those 500 *ADD* in winter, pigs lost roughly 25% of their mass, but this decomposition progress was not reflected in the *TBS* measure, which stayed at 24 as no morphological changes occurred on the remains. Mass loss was therefore able to detect continual differences in decomposition progress between cadaver types that was unable to be detected using the *TBS* method. A similar plateau in *TBS* can be observed in other environments where similar studies have been conducted on decomposing pigs [34, 41]. *TBS* relies on visual morphological changes occurring on a cadaver during decomposition, which occur less frequently in advanced decay [2]. Mass loss on the other hand is a continuous process occurring throughout decomposition, even when limited visual changes in the cadaver are occurring [19]. *TBS* may therefore be unreliable during advanced decay for detecting decomposition changes on cadavers, while direct measures like mass loss are potentially more reliable.

Although differences were observed between mass loss and *TBS* in winter, the data for summer showed a much more linear relationship between mass loss and *TBS*. As decomposition progressed more rapidly in summer, humans and pigs were at similar mass loss and *TBS* values by the end of decomposition, unlike in winter, where mass loss differed at the end of decomposition. Due to this close association, *TBS* can be used as a benchmark to quantify possible mass loss progress in cadavers. For example, based on the model formulas derived from these experiments, we have determined predicted mass loss at different *TBS* intervals (Table 1). Knowing how *TBS* correlates with mass loss could open new avenues of mass loss estimation for other researchers unable to directly measure this variable in their experiments. The relationship between mass loss and *TBS* differs between season and cadaver type as observed here, but also likely differs between habitats and climates; therefore, validation of these relationships is required in other localities [1].

**Table 1:** Estimated mass remaining for different total body scores derived from linear model equations

*TBS*	**Human**	**Human**	**Pig**	**Pig**
	Winter	Summer	Winter	Summer
	Mass remaining	Mass remaining	Mass remaining	Mass remaining
5	100	100	100	93
10	92	81	92	75
15	83	62	78	58
20	74	42	64	40
25	65	23	50	23
30	56	3	36	5

### Implications and conclusion

Decomposition is a complex process influenced by a diverse combination of biotic and abiotic factors. Having a reliable measure of decomposition is key to the development of accurate PMI models for forensic investigators. This study has shown that mass loss can be used as an accurate measurement of decomposition progress. Mass loss can provide more quantitative information about the decomposition progress during advanced decay that is not evident in indirect measures such as *TBS*. Although mass loss is difficult to use in casework due to the often unknown or unreliable starting mass of a cadaver, it is still a valuable tool to incorporate in forensic research and casework. A combination of both direct and indirect decomposition measures, such as mass loss and *TBS*, is therefore recommended to ensure reliable assessment of the decomposition progress. Some estimated values of percentage mass remaining for a range of different *TBS* values have been provided here. These may be useful for estimating mass loss from *TBS* assessments, and further research that tests the robustness of *TBS*-mass loss relationships in a range of other environments is encouraged. This study also highlights the differences in decomposition progress between pigs and humans, suggesting caution when relying solely on pig models for extrapolating to human forensic research, with some differences in mass loss occurring in different seasons.

## Key points

1. Mass loss is seldom used to measure decomposition on human cadavers.
2. Compared with total body score, mass loss is more informative in advanced decay.
3. Mass loss of pigs was more rapid than humans.
